# Patient Portal Messaging for Asynchronous Virtual Care During the COVID-19 Pandemic: Retrospective Analysis

**DOI:** 10.2196/35187

**Published:** 2022-05-05

**Authors:** Ming Huang, Aditya Khurana, George Mastorakos, Andrew Wen, Huan He, Liwei Wang, Sijia Liu, Yanshan Wang, Nansu Zong, Julie Prigge, Brian Costello, Nilay Shah, Henry Ting, Jungwei Fan, Christi Patten, Hongfang Liu

**Affiliations:** 1 Department of Artificial Intelligence and Informatics Mayo Clinic Rochester, MN United States; 2 Mayo Clinic Alix School of Medicine Mayo Clinic Scottsdale, AZ United States; 3 Center for Connected Care Mayo Clinic Rochester, MN United States; 4 Department of Cardiovascular Medicine Mayo Clinic Rochester, MN United States; 5 Center for Clinical and Translational Science Mayo Clinic Rochester, MN United States; 6 Department of Psychiatry and Psychology Mayo Clinic Rochester, MN United States

**Keywords:** patient portal, patient portal message, asynchronous communication, COVID-19, utilization, digital health, healthcare, health care, remote healthcare, virtual care, pandemic

## Abstract

**Background:**

During the COVID-19 pandemic, patient portals and their message platforms allowed remote access to health care. Utilization patterns in patient messaging during the COVID-19 crisis have not been studied thoroughly. In this work, we propose characterizing patients and their use of asynchronous virtual care for COVID-19 via a retrospective analysis of patient portal messages.

**Objective:**

This study aimed to perform a retrospective analysis of portal messages to probe asynchronous patient responses to the COVID-19 crisis.

**Methods:**

We collected over 2 million patient-generated messages (PGMs) at Mayo Clinic during February 1 to August 31, 2020. We analyzed descriptive statistics on PGMs related to COVID-19 and incorporated patients’ sociodemographic factors into the analysis. We analyzed the PGMs on COVID-19 in terms of COVID-19–related care (eg, COVID-19 symptom self-assessment and COVID-19 tests and results) and other health issues (eg, appointment cancellation, anxiety, and depression).

**Results:**

The majority of PGMs on COVID-19 pertained to COVID-19 symptom self-assessment (42.50%) and COVID-19 tests and results (30.84%). The PGMs related to COVID-19 symptom self-assessment and COVID-19 test results had dynamic patterns and peaks similar to the newly confirmed cases in the United States and in Minnesota. The trend of PGMs related to COVID-19 care plans paralleled trends in newly hospitalized cases and deaths. After an initial peak in March, the PGMs on issues such as appointment cancellations and anxiety regarding COVID-19 displayed a declining trend. The majority of message senders were 30-64 years old, married, female, White, or urban residents. This majority was an even higher proportion among patients who sent portal messages on COVID-19.

**Conclusions:**

During the COVID-19 pandemic, patients increased portal messaging utilization to address health care issues about COVID-19 (in particular, symptom self-assessment and tests and results). Trends in message usage closely followed national trends in new cases and hospitalizations. There is a wide disparity for minority and rural populations in the use of PGMs for addressing the COVID-19 crisis.

## Introduction

The COVID-19 pandemic accelerated the adoption of digital and virtual patient care technology as sustainable and scalable parts of health systems. This includes the use of video, audio, and even Health Insurance Portability and Accountability Act–secure portals as a means for patients to remain connected with their providers [1]. Compared with synchronous virtual care such as phone and video visits [2,3], patient portals as secure web-based platforms allow patients to conveniently access information from their electronic health records and asynchronously interact with their providers [4]. Patient portals are becoming increasingly common and give patients unlimited access to their health information (eg, clinical notes, test results, medications, and discharge summaries) from anywhere through an internet connection [5]. A study shows that over 90% of health care organizations (eg, Veterans Administration and Kaiser Permanente) had provided patient portal services to their patients [6]. Convenient access and management of personal health information have been shown to improve patients’ self-management of diseases by increasing awareness of disease knowledge, status, and progress [7]. Additionally, patient portals provide a significant function of portal messaging for asynchronous communication between patients and their providers or care teams on a wide spectrum of tasks such as appointment requests, virtual visits, care management, or mental health issues [8-10].

Millions of nonurgent and non–COVID-19 medical encounters were postponed or cancelled by patients and health systems to reduce the risk of COVID-19 infection during in-person visits and prevent virus spread [11-14]. For continued health care access, most clinic visits had transitioned to web-based platforms for health care access, including COVID-19 diagnosis and treatment [2,15]. Through patient portals, patients could receive educational information on COVID-19 preventive care measures or use web-based triage forms (e-visit) for COVID-19 symptom assessment by an advanced practice provider. If a COVID-19 diagnostic test was recommended (and completed), patients could send and receive portal messages related to their COVID-19 diagnostic tests and test results. Even with a positive test result, patients could communicate with their providers about their COVID-19 symptoms through remote patient monitoring. In addition, the patient portal also provided a COVID-19 symptom self-assessment that patients could use interactively.

In the early stages of the global response, the public health strategy involved isolation for those infected or at risk, reducing social contact to slow the spread, and masking and hand washing to reduce infection risk. However, this unintentionally led to increased feelings of loneliness, reduced access social support, and worsening stress, anxiety, and depressive symptoms [16-18]. Studies indicate that the COVID-19 crisis and resulting economic and social lockdown and isolation had negatively impacted patients’ mental health [19-21]. Patients may be using patient portals to interact with their providers about their mental health conditions to seek support.

Studies on the use of telehealth and patient portal technologies have recently increased during the COVID-19 pandemic [2,15,22]. For example, Patel et al [23] implemented telehealth capabilities for COVID-19 care within their pediatric patient portal and found that weekly telehealth visits subsequently increased 200-fold for children and 90-fold for adolescents. Khairat et al [24] analyzed the use of pediatric tele-urgent care visits via a patient portal at a southeastern health care center and revealed that the use of tele-urgent care visits for pediatric care doubled during the COVID-19 crisis. Portz et al [25] observed a large increase of patient portal utilization for advance care planning. However, very few research studies examined the messaging component of the portal, an important function of patient portals for asynchronous communication between patients and providers, specifically for COVID-19–related care and issues [26]. Thus, in this study, we assessed portal messages associated with COVID-19 generated by patients from February 1 to August 31, 2020, at Mayo Clinic, a large multispecialty academic health system. We summarized reasons for patient utilization of portal messaging for COVID-19–related care such as diagnosis, testing, treatment, scheduling, and mental health support [27]. During this time period, vaccinations for COVID-19 were not available; hence, this topic was not analyzed. In addition, we analyzed patient user demographics with respect to their personal and social factors such as age, gender, race, and geographic location. These findings can provide insight into how patients interacted with the asynchronous portion of the patient portal during the COVID-19 pandemic.

## Methods

### Data Collection and Preprocessing

Mayo Clinic is a large multispecialty academic medical center focused on integrated patient care, education, and research. Mayo Clinic has three main medical sites in Minnesota, Florida, and Arizona and Mayo Clinic Health System (MCHS). MCHS is as a network of community-based medical services and consisted of more than 40 hospitals and clinics in Minnesota, Iowa, and Wisconsin in 2021. Mayo Clinic’s patient portal (Patient Online Services) has been operational since 2010 [28]. We collected over 2 million portal messages generated by patients from the Epic Clarity database between February 1 and August 31, 2020. We filtered the patient-generated messages (PGMs) associated with COVID-19 using relevant keywords (eg, “COVID-19,” “Pandemic,” “Coronavirus,” “SARS-CoV-2,” and “2019-nCoV”) and their synonyms and morphological variations (see Table S1 in the Multimedia Appendix 1). We excluded the PGMs with empty message bodies and the PGMs requested by providers such as messages for preappointment COVID-19 screening and postdischarge COVID-19 symptom checks. We then identified 207,299 portal messages on COVID-19 generated by 102,470 patients from the Epic Clarity database. In addition, the patient portal provided an anonymous COVID-19 self-checker for patients’ self-assessment of COVID-19 symptoms on March 22, 2020. We collected 153,224 PGMs on COVID-19 symptom self-assessment during March 22 to August 31, 2020. Thus, a total of 360,523 PGMs were used for sequential analysis.

### Data Analysis

#### Sociodemographic Characteristics of Patients

We analyzed the distribution of unique patients by age, gender, marriage, ethnicity, race, language, and residence. We excluded anonymous patients who sent messages for COVID-19 symptom self-assessment in the demographic analysis. We conducted a subanalysis comparing three different cohorts within our sample: patients who sent messages related to COVID-19 only (COVID-19 message senders), any patients who sent messages related to any topic (general message senders), and all patients who were active on the portal regardless of whether they composed messages (general patients). Statistical analysis involved chi-square goodness-of-fit tests.

#### PGMs Related to COVID-19

We calculated the daily numbers of total PGMs related to COVID-19 between February 1 and August 31, 2020. The daily numbers would exhibit a week periodicity (typically with a maximum on Monday and a minimum on weekends). Because of this, we calculated their weekly smoothing averages (WSAs). The WSAs displayed a reduction around the holidays (Memorial Day on May 25, 2020, and Independence Day on July 4, 2020); hence, holidays were excluded from the analysis. The daily numbers and WSAs of the PGMs on COVID-19 can approximate the overall utilization of portal messages by the patients for addressing the COVID-19 crisis over time.

#### Messages for COVID-19–Related Care and Other Health Care Issues

We analyzed the PGMs used for assessing COVID-19 symptoms and discussing COVID-19 care plans to understand the message utilization for COVID-19 diagnosis and treatment. We filtered the PGMs on COVID-19 symptom assessment by searching the relevant phrase, “COVID-19 (Coronavirus) Symptom Assessment,” as well as relevant keywords such as “test” and “result” for diagnostic tests and results and “care plan,” “monitoring,” and “interactive care” for care plans (see Table S1 in the Multimedia Appendix 1). We then calculated the daily numbers and WSAs of these PGMs.

In addition, we examined other health care issues caused by COVID-19 reported in the portal messages to understand the impacts of the COVID-19 pandemic on health services and patients. We calculated the number of PGMs explicitly mentioning the phrase “due to COVID-19” and its synonyms to examine patient-reported health care issues caused by the COVID-19 pandemic. We also computed the number of PGMs on COVID-19, which discussed rescheduling or cancelling appointments, mental health, and suicidal ideation using relevant keywords (eg, “cancel” and “reschedule” for appointments, “anxiety” and “depressed” for mental health, and “suicide” for suicidal ideation) and their synonyms to quantify the impact of the COVID-19 pandemic on health services and patients (see Table S1 in the Multimedia Appendix 1).

#### Evaluation

We recruited 2 medical students for annotating the binary code for each studied topic in portal messages: whether a portal message is linked to COVID-19, COVID-19–related care, or other health care issues due to COVID-19. We randomly sampled 1800 portal messages for annotation, and the results are shown in Table S2 in Multimedia Appendix 1. The first 100 portal messages (10 each topic) were sampled and labeled by both annotators and their overall interagreement score is 0.91. After that, the two annotators worked together to complete the rest of the annotation. More specifically, we randomly sampled 100 portal messages on COVID-19 and 900 portal messages not related to COVID-19, which were identified by using the keyword searching for labeling. The precision, recall, and F1-score of the COVID-19 keyword search was over 0.99. We randomly sampled 100 portal messages on COVID-19 linked to each topic such as “isolation.” The F1-scores range from 63.1% to 94.9%, except for “symptom assessment” (100%). The portal message on symptom assessment has a special phrase, “E-Visit Submission: COVID-19 (Coronavirus) Symptom Assessment,” to accurately filter.

### Ethics Approval

No patients were exposed to any intervention. We used the data from the Mayo Clinic Unified Data Platform for analysis. The study was approved by the Mayo Clinic institutional review board (19-002211).

## Results

### Patient Characteristics

Table 1 lists the demographic distribution of patient populations from Epic Clarity database by age, gender, marriage, ethnicity, race, language, and residence. The results of chi-square goodness-of-fit tests among the three patient role groups (COVID-19 message senders, general message senders, and general patients) were listed in Table S2 in Multimedia Appendix 1.

We found that both COVID-19 and general message senders had a significantly different distribution compared to all patients active on the portal (*P*<.001). The distribution of the COVID-19 message senders was also significantly different from that of general message senders in terms of age, gender, marriage, race, and residence (*P*<.001).

**Table 1:** Demographic distribution of patients: COVID-19 message senders, general message senders, and general patients.

Patient demographics		COVID-19 message senders (N=102,470), %	General message senders (N=384,922), %	General patients (N=1,055,319), %
**Age**				
	<18	7.58	8.25	10.67
	18-29	9.80	9.75	10.22
	30-39	13.14	11.81	10.36
	40-49	13.84	12.94	11.45
	50-64	29.11	28.05	24.83
	≥65	26.53	29.20	32.47
**Gender**				
	Female	60.97	58.54	54.37
	Male	39.03	41.46	45.63
**Marital status**				
	Married or has a life partner	63.87	63.22	56.06
	Not married or legally separated	36.13	36.78	43.94
**Ethnicity**				
	Non–Hispanic or Latino	95.84	95.82	95.28
	Hispanic or Latino	4.16	4.18	4.72
**Race**				
	White	92.73	92.40	90.84
	Asian	2.44	2.42	2.28
	Black or African American	2.11	2.44	3.40
	American Indian or Alaska Native	0.37	0.37	0.41
	Native Hawaiian or Pacific Islander	0.09	0.10	0.12
	Other	2.26	2.28	2.94
**Language**				
	English	99.09	99.06	97.56
	Arabic	0.12	0.14	0.25
	Spanish	0.34	0.37	1.03
	Other	0.45	0.44	1.16
**Residential area**				
	Urban	74.77	70.41	61.02
	Rural	25.23	29.59	38.98

More than half (>55%) of patients were in the age groups of 50-64 years and ≥65 years. The proportion of patients in the age ranges of 30-39 years, 40-49 years, and 50-59 years was observed to have increased when looking at general portal users to general message senders to COVID-19–specific message senders. Meanwhile, the proportion of message senders in the age groups of <18 years and ≥65 years was the lowest in the COVID-19 message sender cohort.

More than half (>54%) of the patients were female and were married or had a life partner. The proportion of female patients (61% vs 54%) and married patients (64% vs 56%) also was highest in the COVID-19 message sender cohort when compared to the entire active portal user cohort. More than 90% of patients were of non–Hispanic or Latino ethnicity, White race, and spoke English. This proportion was also highest in the COVID-19 message sender cohort and lowest in the general portal cohort.

At least 61% of all patients assessed lived in the urban area. The percentage of urban patients increased to 70% among general message senders and 75% among COVID-19 message senders.

### PGMs Related to COVID-19

**Figure 1:**
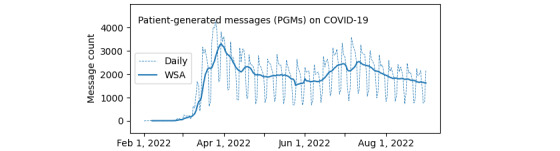
Daily numbers and weekly smoothing averages (WSAs) of patient-generated messages (PGMs) related to COVID-19.

We illustrated the daily numbers and WSAs of PGMs on COVID-19 in Figure 1. The WSA of PGMs on COVID-19 started to increase at the end of February and quickly peaked at 3303.43 messages per week on March 30. Sequentially, the WSA of PGMs on COVID-19 decreased to a local minimum of 1750.29 messages per week on June 5. The WSA of PGMs on COVID-19 reached a local maximum of 2624.57 messages per week on July 12. This July peak was 79.45% of the March peak’s volume. The WSA of PGMs on COVID-19 displayed a consistently declining trend over time after this point. Table 2 lists the numbers and proportions of PGMs for COVID-19–related care and other health care issues.

**Table 2:** Patient-generated messages (PGMs) related to COVID-19–related care and other health care issues caused by COVID-19.

Category		PGMs on COVID-19 (N=360,523), n (%)
**COVID-19–related care**		
	Self-checker	153,224 (42.50)
	e-Visit	4619 (1.28)
	Tests and results	111,183 (30.84)
	Care plan	3844 (1.07)
**Other issues caused by the COVID-19 pandemic**		
	General issues	13,333 (3.70)
	Postponement	26,924 (7.47)
	Cancellation	19,000 (5.27)
	Anxiety	21,413 (5.94)
	Depression	3673 (1.02)
	Suicidal ideation	288 (0.08)

### Messages for COVID-19–Related Care and Other Health Care Issues

**Figure 2:**
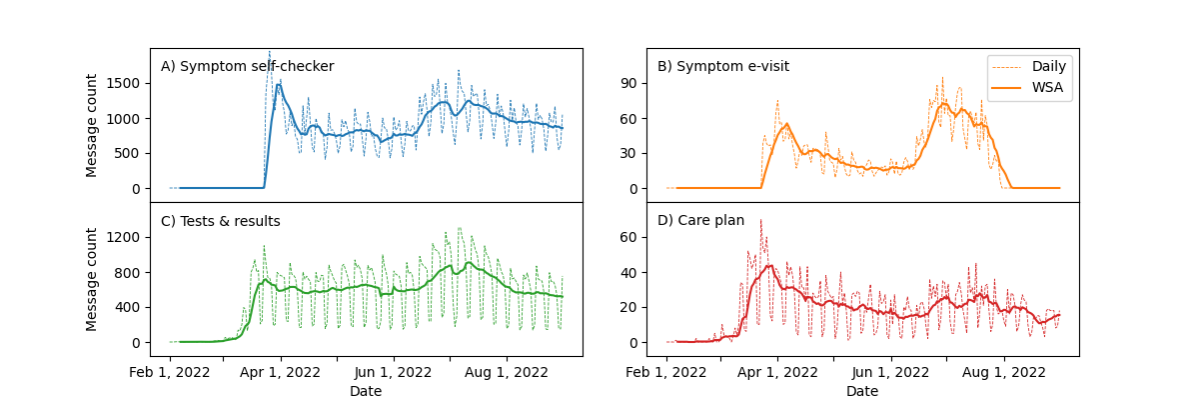
Daily numbers and weekly smoothing averages (WSAs) of patient-generated messages (PGMs) regarding COVID-19–related care (diagnosis and treatment): (A) COVID-19 symptom assessment via self-checker, (B) COVID-19 symptom assessment by providers via e-visits, (C) discussions regarding COVID-19 tests and results, and (D) care plans.

Figure 2 depicts the daily numbers and WSAs of PGMs for COVID-19 symptom assessment via the self-checker (Figure 2A), COVID-19 symptom assessment via e-visits (Figure 2B), discussing the COVID-19 diagnostic tests and results (Figure 2C), and the care plan (Figure 2D). The top message concepts for COVID-19–related care were COVID-19 symptom assessment via the self-checker (42.50%) and COVID-19 tests and results (30.84%). The percentage of PGMs on COVID-19 symptom assessment associated with e-visits or the COVID-19 care plan was approximately 1%. Owing to the low use of COVID-19 symptom assessment via e-visits, the patient portal stopped the e-visit service for COVID-19 symptom assessment on August 1, 2020.

Similar to the total PGMs on COVID-19 in Figure 1, the PGMs on COVID-19 symptom assessment via self-checker and COVID-19 tests and results had analogous dynamic patterns: two peaks in late March or early April and late June or early July. These fluctuations were consistent with the surge in newly confirmed COVID-19 cases in the United States and in Minnesota (see Figure S1 in Multimedia Appendix 1) [11]. The PGMs for COVID-19 symptom assessment via e-visits had two similar peaks and surges before the termination of the service. The trend of PGMs related to COVID-19 care plans paralleled trends in newly hospitalized cases and deaths in the United States and in Minnesota (see Figure S1 in the Multimedia Appendix 1) [11].

**Figure 3:**
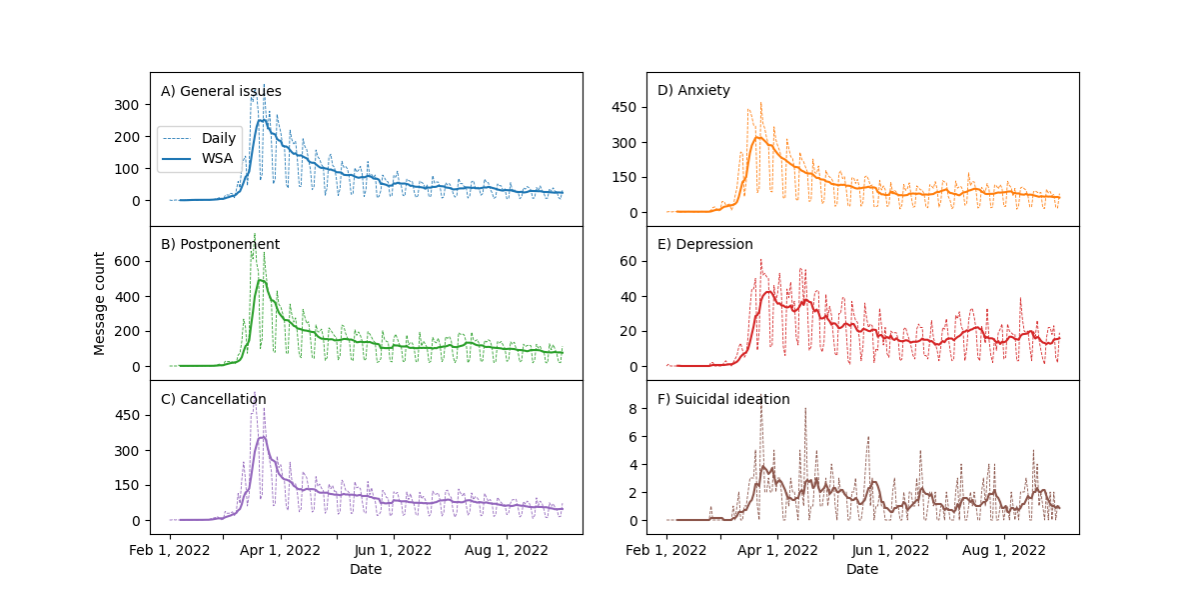
Daily numbers and weekly smoothing averages (WSAs) of patient-generated messages (PGMs) regarding COVID-19–related other health care issues: (A) general issues due to COVID-19, (B) postponement, (C) cancellation, (D) anxiety, (E) depression, and (F) suicidal ideation.

Table 2 shows that the top message usage for other health care issues caused by COVID-19 was related to appointment postponement (7.47%), anxiety (5.94%), and appointment cancellation (5.27%). Among the studied mental health issues, the number of relevant PGMs decreased as the severity of health issues increased: anxiety (5.94%), depression (1.02%), and suicidal ideation (0.08%). Figure 3 depicts the numbers of PGMs related to COVID-19, which mentioned general issues (Figure 3A), appointment postponement (Figure 3B), appointment cancellation (Figure 3C), anxiety (Figure 3D), depression (Figure 3E), and suicidal ideation (Figure 3F). The curves in Figure 3 show a similar trend over time: the number of PGMs on COVID-19 started to increase in early March and quickly peaked within 2-3 weeks. However, after March 30, the number of PGMs on COVID-19 constantly decreased, although smaller upward fluctuations occurred from July to August in some curves; for example, such as PGMs for postponement, anxiety, and depression. These fluctuations paralleled those observed in PGM use for COVID-19 care plans, as shown in Figure 2D.

## Discussion

### Principal Findings

The COVID-19 pandemic and subsequent public health mitigation strategies, including stay-at-home orders and business restrictions, substantially impacted delivery of health care services. As the COVID-19 pandemic progressed in the United States, and specifically in Minnesota, newly confirmed cases had two peaks during February 1 and August 31, 2020, owing to the initial outbreak and late termination of stay-at-home orders. We observed similar dynamic patterns in PGMs on COVID-19, particularly, COVID-19 diagnosis and treatment, suggesting that patients actively used the portal messaging for addressing their concerns regarding the COVID-19 crisis [23]. Another previous study [26] was consistent with our findings, as they analyzed messages in an ambulatory practice network and determined that their inbox message usage patterns were also consistent with national trends. Patients sent portal messages mainly for COVID-19 symptom self-assessment and discussing COVID-19 tests and results. It appeared that patients preferred symptom self-assessment to e-visits for symptom assessment given the utilization rates. Thus, analyzing PGMs on COVID-19 symptom assessment via self-checker before diagnostic testing could serve as a timely surveillance of COVID-19. Prior work by Denis et al [29] utilized this association when developing a self-assessment web-based app to assess trends of the COVID-19 pandemic in France. We also determined that the PGMs related to COVID-19 care plans followed trends in newly hospitalized cases and deaths. The second relative maximum of PGMs on the COVID-19 care plan after May suggests a decline of COVID-19 risk during that time period.

Our findings also indicated that patients used the portal to report feelings of anxiety and depression about their existing medical conditions and potential contagious risks due to COVID-19 and seek support from their providers. Similar mental health concerns increased in the general population, according to a study of Twitter data, which showed an increased in tweets expressing mental health concerns due to infection risk and isolation strategies in the early stages of the pandemic [30]. After examining 100 samples of related portal messages, we found that patients often reported worry about worsening of their current illnesses without their typical in-person follow-up as well as concern for COVID-19 infection risk during their visits to the clinics or hospitals. Some patients were also nervous about falling ill because they were not able to afford health care services owing to loss of jobs and health insurances [31]. Under such stressors, some patients reported depressive symptoms, and a few indicated suicidal thoughts and requested medical advice from their providers [32,33]. Although the number of PGMs on these issues rose substantially at the beginning of March, the relevant PGM count was progressively declining over time after April. The trends of PGMs related to the mental health concerns were consistent with those of PGMs related to COVID-19 care plans. We speculate the findings on the decline in overall mental health issues due to COVID-19 among patient portal users may result from a reduction in COVID-19 risk, eventual management of medical conditions, adaption to mitigation activities, or support from their caregivers. For example, a study on web-based search behavior for mental health concerns in the United States demonstrated a significant flattening of the curve for searches for anxiety and suicidal ideation after implementation of stay-at-home orders in certain states [34].

After analyzing PGMs related to COVID-19 and unique patient senders from the Epic Clarity system (see Figure S2 in Multimedia Appendix 1), we found a low messaging rate per patient and a strong correlation between message count and unique patient count, which suggests that the volume of unique patients mainly contribute to the intensive utilization of portal messaging. Demographic analysis of patient populations shows a significant difference in the distribution of patient populations between general message senders and general portal users. Compared to general portal users, the frequent message senders were more so middle-aged adults (30-64 years old), female, married (or with life partners), non–Hispanic or Latino, White, English speakers, or urban residents. This proportion was ever more pronounced in COVID-19 message senders. Middle-aged (or female, married, White, and urban) patients were more inclined to use patient portals for addressing their issues regarding COVID-19. This phenomenon is interesting given the fact that the COVID-19 pandemic disproportionately affected racial minorities and rural populations, who are particularly vulnerable to severe outcomes of COVID-19 [35,36]. This may be a consequence of the patient population of the institution or rather that patient portal has some inherent bias toward more health-literate patient populations, which may not be the same populations as negatively affected by the pandemic.

Telehealth, including the use of patient portals, is transforming the delivery of health care [2]. An unintended effect of the growth of patient portal messages may be an increased workload for providers. A previous study reported that providers occasionally needed to reply to messages sent by patients after work hours in order to ensure timely response. Newer delivery models will need to properly distribute the communication load for better efficiency and avoid provider burnout [26,37]. Some health care systems will probably face this challenge of managing increasing volumes of patient messages in the near future [38]. These health care systems will require new billing models and practice metrics, or additional ancillary infrastructure, including support staff, to accommodate this growing trend of asynchronous communication. Evolving technologies in artificial intelligence and natural language processing tools may even be considered as technological support for care teams in secure messaging [39,40]. In addition, the observed disparities in use of remote patient care among these populations warrants attention from providers and researchers on designing inclusive as well as innovative solutions to achieve equity in health care service delivery [41].

### Limitations

There are several limitations to our study. First, the patient portal messages were collected at Mayo Clinic, a multispecialty academic medical center. The collected data might not be representative of different clinical settings or patient populations in other areas of the country. Second, keyword searching was carried out to identify patient portal messages associated with COVID-19, COVID-19–related care, and other health care issues due to COVID-19. Although the keyword sets cover a large number of relevant keywords, synonyms, and morphological variations, they may not be totally comprehensive; hence, bias could exist in our results. We are developing robust detection algorithms based on state-of-art deep learning techniques to accurately identify interesting health topics in portal messages during the pandemic. Finally, we investigated patient portal messages in the early stage of the COVID-19 pandemic. The analysis of patient portal messages in the following time or later stages of the pandemic is beyond the scope of this study but represents an important area for further exploration.

### Conclusions

During the COVID-19 pandemic, patient portal utilization increased to address questions and concerns about the COVID-19 pandemic, revolving mainly around symptom self-assessment, tests, and results. The increased usage statistics for COVID-19 indicates the patient portal was a valuable web-based platform for patients to remotely discuss COVID-19 diagnosis and treatment as well as seek support for other health care issues impacted by the pandemic. The volume of PGMs on COVID-19–related care fluctuated as the pandemic developed. After initial increase in March, the PGMs regarding other health care issues such as appointment cancellations and anxiety about disease progression exhibited a declining trend. We observed differences in patient demographics between general portal users, general message senders, and COVID-19–specific message senders, mainly that the majority demographic took on a larger proportion of COVID-19 messages. There is still great potential to increase PGM engagement for minority populations and rural communities with regard to the COVID-19 pandemic. Time-series analysis of portal messages could offer us a timely surveillance of COVID-19 and its impacts on patients to improve patient-centered care related to the COVID-19 crisis.

## Appendix

Multimedia Appendix 1 Table S1 Keywords used to filter relevant portal messages; Table S2. Performance of Keyword searching methods; Table S3 Chi-squared goodness-of-fit test between patient role groups; Figure S1 Newly confirmed COVID-19 cases, hospitalized cases, and deaths in the US and Minnesota; Figure S2 Daily numbers and weekly smoothing averages (WSAs) of patient-generated messages (PGMs), unique patient senders, and messages per patient from the Epic Clarity database.

## Abbreviations

MCHS: Mayo Clinic Health System
PGM: patient-generated message
WSA: weekly smoothing average
