# Supplementary material for: Patient Portal Messaging for Asynchronous Virtual Care During the COVID-19 Pandemic: Retrospective Analysis
Source: JMIR Hum Factors. 2022 May 5;9(2):e35187. doi: 10.2196/35187 (PMC9084445; doi:10.2196/35187)
Supplement: Multimedia Appendix 1 [file humanfactors_v9i2e35187_app1.docx]

**Patient Asynchronous Response to Coronavirus Disease 2019 (COVID-19): A Retrospective Analysis of Patient Portal Messages**

Ming Huang PhD^1^, Aditya Khurana MD^2^, George Mastorakos MD^2^,

Andrew Wen MS^1^, Huan He PhD^1^, Liwei Wang PhD^1^, Sijia Liu PhD^1^, Yanshan Wang PhD^1^, Nansu Zong^1^, Julie E Prigge MA^3^, Brian A Costello MD^3^, Nilay D Shah PhD^1^, Henry H Ting MD, MBA^1,4,^,Jung-wei Fan PhD^1^, Christi A Patten PhD^5,6^, and Hongfang Liu PhD^1^

^1^Department of Artificial Intelligence and Informatics, Mayo Clinic, Rochester, MN, USA

^2^Mayo Clinic Alix School of Medicine, Mayo Clinic, Scottsdale, AZ, USA

^3^Center for Connected Care, Mayo Clinic, Rochester, MN, USA

^4^Department of Cardiovascular Medicine, Mayo Clinic, Rochester, MN, USA

^5^Center for Clinical and Translational Science, Community Engagement Program, Mayo Clinic, Rochester, MN, USA

^6^Department of Psychiatry and Psychology, Mayo Clinic, Rochester, MN, USA

**Corresponding author**:

Hongfang Liu

Department of Artificial Intelligence and Informatics

Mayo Clinic

Rochester, MN, United States

E-mail: Liu.Hongfang@mayo.edu

Telephone: 507-293-0057

**Table S1** Keywords used to filter relevant portal messages

| Category | Keywords |
| --- | --- |
| COVID-19 | Coronavirus, Corona virus, Pandemic, COVID, COVID-19, 2019-nCoV, SARS-CoV |
| Tests and results | Test, Result, Polymerase chain reaction, PCR, Serology |
| Care plan | Care plan, care of plan, Monitoring, Interactive Care Plan, ICP, Remote Patient Monitoring, RPM |
| General issue | Given, Considering, d/t, Since, Because, Due to, Because of, Thanks to, In light of, In view of, In consideration of, On account of, As a result of, By reason of, Caused by COVID |
| Cancellation | Cancel |
| Postponement | Reschedule, Postpone, Hold off, Held off, Suspend, Put on hold, Defer, Delay |
| Anxiety | Anxiety, Anxious, Nervous, Worry, Worried, Uneasy, Overwhelm, Jittery, Fear, Panic, Fright, Dread, Afraid, Scare, Terror, terrify, Petrif, Alarmed |
| Depression | Depress, Sad, Sadly, Sadness, Sadden, Dishearten, Dismay, Low mood, Hopeless, Desperate |
| Suicidal ideation | Suicide, Kill myself, Killing myself |

**Table S2.** Performance of Keyword searching methods

| Category | Precision | Recall | F1-score |
| --- | --- | --- | --- |
| COVID-19 | 100.0 | 99.0 | 99.5 |
| Symptom assessment | 100.0 | 100.0 | 100.0 |
| Tests & results | 53.0 | 77.9 | 63.1 |
| Care plan | 90.0 | 94.7 | 92.3 |
| General issues | 95.0 | 79.2 | 86.4 |
| Postponement | 96.0 | 85.0 | 90.1 |
| cancellation | 96.0 | 85.7 | 90.6 |
| Anxiety | 95.0 | 90.5 | 92.7 |
| Depression | 93.0 | 96.9 | 94.9 |

**Table S3** Chi-squared goodness-of-fit test between patient role groups

| Pair of Patient Populations | Demographics | P-value | Chi-square Statistics | Critical Value |
| --- | --- | --- | --- | --- |
| General Message Senders V.S. All Patients | Age | 0 | 6596.09 | 12.59 |
|  | Area | 0 | 14250.69 | 5.99 |
|  | Ethnicity | 6.39E-56 | 248.21 | 5.99 |
|  | Gender | 0 | 2696.58 | 5.99 |
|  | Language | 0 | 3670.26 | 9.49 |
|  | Marriage | 0 | 7994.25 | 5.99 |
|  | Race | 0 | 1775.84 | 12.59 |
| COVID-19 Message Senders V.S. All Patients | Age | 0 | 4076.23 | 12.59 |
|  | Area | 0 | 8133.59 | 5.99 |
|  | Ethnicity | 6.32E-17 | 69.87 | 5.99 |
|  | Gender | 0 | 1799.04 | 5.99 |
|  | Language | 1.92E-220 | 1018.31 | 9.49 |
|  | Marriage | 0 | 2530.69 | 5.99 |
|  | Race | 2.13E-153 | 720.17 | 12.59 |
| COVID-19 Message Sender V.S. General Message Senders | Age | 1.96E-119 | 563.03 | 12.59 |
|  | Area | 4.89E-205 | 933.59 | 5.99 |
|  | Ethnicity | 0.82 | 0.05 | 5.99 |
|  | Gender | 3.39E-56 | 249.47 | 5.99 |
|  | Language | 0.22 | 4.46 | 9.49 |
|  | Marriage | 1.86E-05 | 18.33 | 5.99 |
|  | Race | 4.94E-09 | 47.30 | 12.59 |


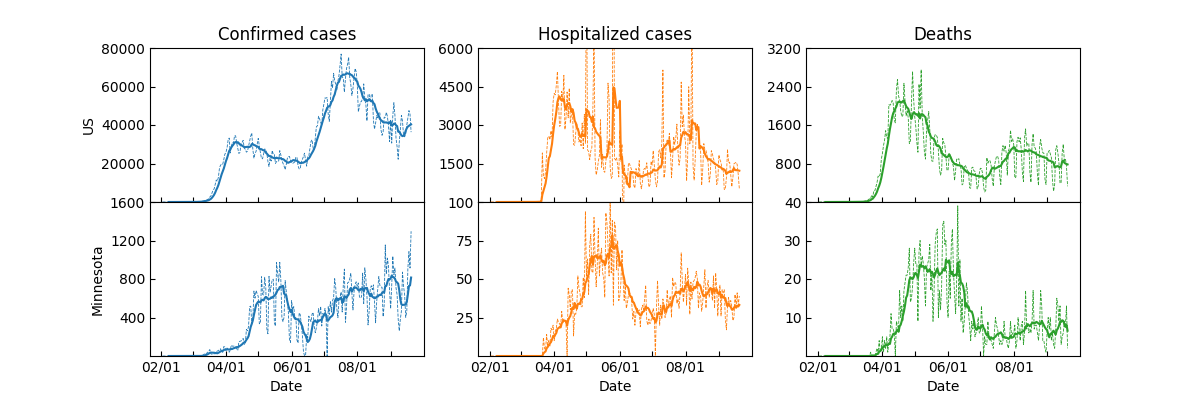


**Figure S1** Newly confirmed COVID-19 cases, hospitalized cases, and deaths in the US and Minnesota


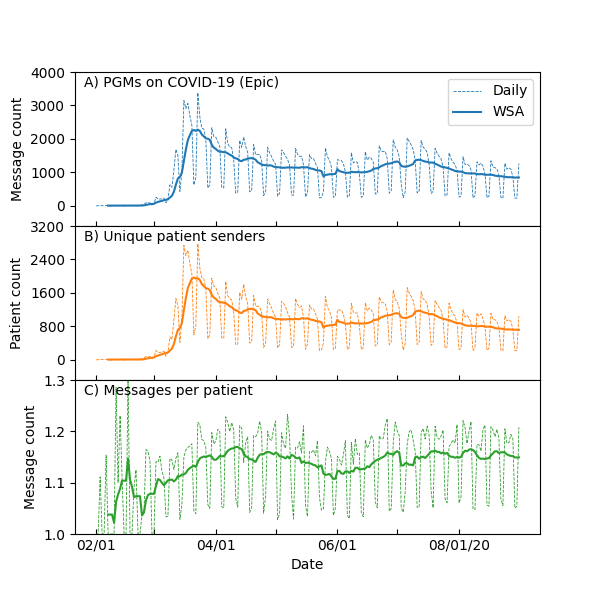


**Figure S2** Daily numbers and weekly smoothing averages (WSAs) of patient-generated messages (PGMs), unique patient senders, and messages per patient from the Epic Clarity database
